# Detection of Zika Virus in April 2013 Patient Samples, Rio de Janeiro, Brazil

**DOI:** 10.3201/eid2312.171375

**Published:** 2017-12

**Authors:** Sonia R. Lambert Passos, Maria A. Borges dos Santos, José Cerbino-Neto, Sibelle N. Buonora, Thiago M.L. Souza, Raquel V.C. de Oliveira, Alexandre Vizzoni, Giselle Barbosa-Lima, Yasmine R. Vieira, Marcondes Silva de Lima, Yara H. M. Hökerberg

**Affiliations:** Oswaldo Cruz Foundation, Rio de Janeiro, Brazil (S.R.L. Passos, T.M.L. Souza);; Estácio de Sá University, Rio de Janeiro (S.R.L. Passos, Y.H.M. Hökerberg);; Fundação Oswaldo Cruz Escola Nacional de Saúde Pública, Rio de Janeiro (M.A. Borges dos Santos);; Evandro Chagas National Institute of Infectious Diseases, Rio de Janeiro (J. Cerbino-Neto, S.N. Buonora, R.V.C. de Oliveira, A. Vizzoni, G. Barbosa-Lima, Y.R. Vieira, M.S. de Lima, Y.H.M. Hökerberg);; Universidade Federal do Rio de Janeiro, Rio de Janeiro (S.N. Buonora)

**Keywords:** Zika virus infection, Zika virus, ZIKV, epidemiological monitoring, communicable diseases, emerging, vector-borne infections, viruses, Brazil, dengue outbreak, zoonoses

## Abstract

We tested 210 dengue virus‒negative samples collected from febrile patients during a dengue virus type 4 outbreak in Rio de Janeiro in April 2013 and found 3 samples positive for Zika virus. Our findings support previously published entomological data suggesting Zika virus was introduced into Brazil during October 2012–May 2013.

In 2016, Brasil et al. (*1*), on the basis of a large number of suspected (n = 364) and laboratory-confirmed (n = 119) cases, reported the first Zika virus outbreak in Rio de Janeiro, with peak transmission in May 2015. Reports confirming Zika virus infection by reverse transcription PCR (RT-PCR) indicated the virus was present earlier in Rio Grande do Norte, Brazil, in October 2014 (*2*) and in Bahia, Brazil, in May 2015 (*3*). These cases were thought to be the first to occur in humans in Brazil and to correspond with the first occurrences of presumptive vectorborne transmission of Zika virus in the continental Americas.

By August 2015, Zika virus infection had been confirmed in 13 states of Brazil (Bahia, Rio Grande do Norte, São Paulo, Alagoas, Pará, Roraima, Rio de Janeiro, Maranhão, Pernambuco, Ceará, Paraíba, Paraná, and Piauí), some of which were located >2,500 miles apart (*4*). Because Zika virus circulation can occur simultaneously with dengue virus (DENV) in regions plagued by *Aedes aegypti* mosquitoes, we used frozen serum samples previously collected during a DENV type 4 (DENV-4) outbreak to investigate whether co-circulation might have been occurring before reported cases.

We evaluated 210 samples collected from patients (median age 36.6 years) with acute febrile syndrome who visited an acute healthcare facility in Tijuca, a middle-class district in the northern zone of Rio de Janeiro, Brazil, during a DENV-4 outbreak occurring March‒May 2013. All samples tested negative for DENV RNA by RT-PCR and DENV nonstructural protein 1 by Platelia Dengue NS1 Ag ELISA (BioRad Laboratories, Marnes-la-Coquette, France) (*5*).

In June 2017, we performed a molecular test to rapidly detect Zika virus in previously frozen acute-phase samples. We extracted viral RNA from 200-µL samples by using the QIAamp Viral RNA Mini Kit (QIAGEN, Valencia, CA, USA) according to the manufacturer’s instructions. We performed quantitative RT-PCR (qRT-PCR) with the QuantiNova Probe RT-PCR Kit (QIAGEN) in a Rotor-Gene Q Sequence Detection System (QIAGEN) using 25-µL reaction mixtures containing 5 µL of RNA template. We used primers, probes, and cycling conditions for Zika virus detection recommended by the Centers for Disease Control and Prevention (*6*). Samples suspected positive (defined as having a cycle threshold <38) were retested in triplicate, and consistently positive samples were confirmed by repeating RNA extraction and qRT-PCR in duplicate.

Of the 210 samples, 21 tested positive by qRT-PCR and were thus suspected positive for Zika virus; 4 of 21 tested positive for Zika virus RNA in triplicate qRT-PCR reactions. However, 1 of the 4 also tested positive by Panbio Dengue IgM Capture ELISA (Standard Diagnostics Inc., Yongin, South Korea). We confirmed that the other 3 samples (2 from men and 1 from a woman) were positive for Zika virus genome after repetition of RNA extraction and qRT-PCR.

Zika virus‒positive patients were young (18, 25, and 26 years of age), lived in Tijuca, had low-grade fever (1‒2 days) during acute disease, and had no underlying conditions. Their travel histories were not available. All patients reported prostration, myalgia, arthralgia, headache, retro-orbital pain, and nausea (Table). None reported rash or hemorrhages. Hematocrit levels were 40%‒45%, platelet counts 2.19‒3.53 × 10^5^/µL, and leukocyte counts 4.4‒19.8 × 10^3^ cells/µL.

**Table:** Distribution of clinical signs and symptoms among 3 patients retrospectively identified as having Zika virus infection, Rio de Janeiro, Brazil, 2013*

No. patients, sign or symptom	Patient A	Patient B	Patient C
3 patients			
Arthralgia†	Yes	Yes	Yes
Fever†, no. days	Yes, 1	Yes, 2	Yes, 1
Headache	Yes	Yes	Yes
Myalgia	Yes	Yes	Yes
Nausea	Yes	Yes	Yes
Prostration	Yes	Yes	Yes
Retroorbital pain	Yes	Yes	Yes
2 patients			
Adenomegaly	Yes	Yes	No
Chills	Yes	No	Yes
Dizziness	Yes	Yes	No
Low back pain	No	Yes	Yes
Taste alteration	Yes	Yes	No
Vomiting	Yes	No	Yes
1 patient			
Anorexia	No	No	Yes
Cold extremities	Yes	No	No
Cough	Yes	No	No
Dyspnea	No	Yes	No
Eye congestion	No	Yes	No
Eye redness perception†	No	Yes	No
Hemoconcentration	No	No	Yes
Hoarseness	No	Yes	No
Leukopenia	No	Yes	No
Oropharyngeal pain	Yes	No	No
Otalgia	No	Yes	No
Pruritus	No	Yes	No
Thready pulse	Yes	No	No

*No patients had exanthema. †Signs and symptoms considered in Brazilian Ministry of Health’s definition for suspected Zika virus infection (http://portalsaude.saude.gov.br/index.php/descricao-da-doenca-zika).

Zika virus dissemination beyond Asia and Africa occurred after the 2007 epidemic in Micronesia (*6*) and, in particular, after the 2013–2014 outbreak in French Polynesia, which involved a large number of symptomatic patients and patients with severe disease, with some having neurologic syndromes (*7*). Brasil et al. (*1*) stated that the phylogenetic analysis of cases in Rio de Janeiro supports the hypothesis that Zika virus was introduced into the city in August 2014, possibly during the International Va’a Federation World Sprint Championship canoe race, which included teams from 4 Zika virus‒endemic countries of the Pacific region. Faria et al. (*7*) used viral genome analyses of the southeastern Asia and Pacific founder lineage to estimate that Zika virus was present in Brazil by February 2014; these authors also suggested that the northeast region of Brazil was the initial virus dissemination point. Massad et al. (*8*) used mathematical models and concluded that Zika virus was most likely introduced into Brazil by infected travelers arriving during October 2013‒March 2014.

However, our findings suggest that Zika virus had already been circulating in Rio de Janeiro since April 2013, consistent with the report by Metsky et al. (*9*) stating that Zika virus had been circulating undetected in multiple regions for many months before the initial case reports. This view is also supported by entomological data from Ayllón et al. (*10*), who used a surveillance program involving field-trapped mosquitoes to perform genetic analyses of mosquitoborne viruses found in Rio de Janeiro during February 2014‒June 2016. Their results suggest that Zika virus was probably already in circulation in Rio de Janeiro during May–November 2013, introduced multiple times from different in-country sources, and that the virus was introduced into the Americas via Brazil during October 2012‒May 2013 (*10*).
